# Association of the fat mass index with hepatic steatosis and fibrosis: evidence from NHANES 2017–2018

**DOI:** 10.1038/s41598-024-57388-1

**Published:** 2024-03-23

**Authors:** Lihe Liu, Jiaxi Lin, Minyue Yin, Lu Liu, Jingwen Gao, Xiaolin Liu, Jinzhou Zhu, Airong Wu

**Affiliations:** 1 Department of Gastroenterology, The First Affiliated Hospital of Soochow University, Suzhou, 215006 China; 2 Suzhou Clinical Center of Digestive Diseases, Suzhou, 215006 China; 3 Department of Gastroenterology, Beijing Friendship Hospital, Capital Medical University, Beijing, 100050 China; 4 Key Laboratory of Hepatosplenic Surgery, Ministry of Education, The First Affiliated Hospital of Harbin Medical University, Harbin, 150001 China

**Keywords:** Diseases, Medical research

## Abstract

Limited population-based studies discuss the association between fat mass index (FMI) and the risk of liver diseases. This investigation utilized data from the National Health and Nutrition Examination Survey (NHANES) to examine the linkage between the FMI and liver conditions, specifically steatosis and fibrosis. The study leveraged data from NHANES’s 2017–2018 cross-sectional study, employing an oversampling technique to deal with sample imbalance. Hepatic steatosis and fibrosis were identified by vibration-controlled transient elastography. Receiver operating curve was used to assess the relationship of anthropometric indicators, e.g., the FMI, body mass index (BMI), weight-adjusted-waist index (WWI), percentage of body fat (BF%), waist-to-hip ratio (WHR), and appendicular skeletal muscle index (ASMI), with hepatic steatosis and fibrosis. In this study, which included 2260 participants, multivariate logistic regression models, stratified analyses, restricted cubic spline (RCS), and sharp regression discontinuity analyses were utilized. The results indicated that the WHR and the FMI achieved the highest area under the curve for identifying hepatic steatosis and fibrosis, respectively (0.720 and 0.726). Notably, the FMI presented the highest adjusted odds ratio for both hepatic steatosis (6.40 [4.91–8.38], *p* = 2.34e−42) and fibrosis (6.06 [5.00, 7.37], *p* = 5.88e−74). Additionally, potential interaction effects were observed between the FMI and variables such as the family income-to-poverty ratio, smoking status, and hypertension, all of which correlated with the presence of liver fibrosis (*p* for interaction < 0.05). The RCS models further confirmed a significant positive correlation of the FMI with the controlled attenuation parameter and liver stiffness measurements. Overall, the findings underscore the strong link between the FMI and liver conditions, proposing the FMI as a potential straightforward marker for identifying liver diseases.

## Introduction

Hepatic steatosis is commonly observed in patients with chronic liver illnesses of various aetiologies, including alcoholic liver disease and viral hepatitis. Additionally, its incidence is on the rise in tandem with the global epidemic of nonalcoholic fatty liver disease (NAFLD)^1–4^. Recent research has brought attention to the influence of steatosis in accelerating the advancement of NAFLD to fibrosis and ultimately cirrhosis. Liver fibrosis is widely recognized as a risk factor for the development of liver cirrhosis and hepatocellular carcinoma^5^. Therefore, the prompt identification of hepatic steatosis and fibrosis is of great significance in the provision of efficient therapy for chronic liver disease before it progresses to an irreversible state.

The gold standard for assessing hepatic steatosis and fibrosis is liver biopsy for histology. Nevertheless, its limited usage can be attributed to its intrusive nature, poor repeatability, and potential for serious complications. Imaging techniques offer alternatives for the characterization of noninvasive steatosis and fibrosis by indicating changes in the physical properties of liver tissue with the development of fatty liver and liver fibrosis^6,7^. Vibration-controlled transient elastography (VCTE) represents a noninvasive tool with the ability to simultaneously assess hepatic steatosis and fibrosis^8^.

While body mass index (BMI) is widely utilized in clinical settings to evaluate body composition, its ability to accurately distinguish between muscle and adipose tissues is limited^9^. Due to its speed, simplicity, and minimal radiation exposure, dual-energy X-ray absorptiometry (DXA) is a largely acknowledged method for determining body composition^10^. However, there are few investigations on the correlation among the fat mass index (FMI), percentage of body fat (BF%), as measured by DXA, and the presence of hepatic steatosis and fibrosis.

The aim of this study was to evaluate the association of anthropometric indicators, including the FMI, BMI, weight-adjusted-waist index (WWI), BF%, waist-to-hip ratio (WHR), and appendicular skeletal muscle index (ASMI), with hepatic steatosis and fibrosis using National Health and Nutrition Examination Survey (NHANES) data.

## Results

### Baseline characteristics

The demographic characteristics of the 2260 participants (non-steatosis: 1081 vs. steatosis: 1179; non-fibrosis: 2090 vs. fibrosis: 170) are summarized in Table 1. The individuals involved in this study had an average age of 38.20 ± 12.57 years; 47.74% were men, and 52.26% were women. Among patients diagnosed with steatosis, it was found that 11% had a BMI within the normal range, 32% were classified as overweight, and the majority, 57%, were categorized as obese. In contrast to participants without hepatic steatosis and fibrosis, those with fatty liver disease had a greater propensity towards being male, being older, and having elevated ALT, AST, LDH, and TG levels. Statistical differences were observed in the distribution of age, history of hypertension and diabetes, and HDL, ALB, GLB, ALT, AST, LDH, and TG levels (*p* values < 0.05). In addition, the balanced fibrosis dataset (non-fibrosis: 2090 vs. fibrosis: 2136) was used in the investigation of fibrosis in this study (Figure S1).

**Table 1:** Demographic and clinical characteristics of the participants divided by hepatic steatosis and fibrosis.

Characteristic	Non-steatosis, N = 1081*	Steatosis, N = 1179*	p-value^#^	Non-fibrosis, N = 2090*	Fibrosis, N = 170*	*p*-value^#^
Race			< 0.001			0.924
Mexican American	125(12%)	250(21%)		344(16%)	31(18%)	
Other Hispanic	107(9.9%)	122(10%)		212(10%)	17(10%)	
Non-Hispanic White	361(33%)	341(29%)		648(31%)	54(32%)	
Non-Hispanic Black	226(21%)	189(16%)		383(18%)	32(19%)	
Other Race	262(24%)	277(23%)		503(24%)	36(21%)	
Gender			< 0.001			0.059
Male	468(43%)	611(52%)		986(47%)	93(55%)	
Female	613(57%)	568(48%)		1104(53%)	77(45%)	
BMI (kg/m^2^**)**			< 0.001			< 0.001
Normal (< 25)	579(54%)	135(11%)		695(33%)	19(11%)	
Overweight (25–30)	332(31%)	373(32%)		682(33%)	23(14%)	
Obese (> 30)	170(16%)	671(57%)		713(34%)	128(75%)	
Education Level			0.022			0.069
Less than high school	150(14%)	205(17%)		320(15%)	35(21%)	
High school or above high school	931(86%)	974(83%)		1770(85%)	135(79%)	
Hypertension	130(12%)	346(29%)	< 0.001	410(20%)	66(39%)	< 0.001
Diabetes	31(2.9%)	169(14%)	< 0.001	151(7.2%)	49(29%)	< 0.001
Smoking	341(32%)	432(37%)	0.011	708(34%)	65(38%)	0.249
Excessive Alcohol Consumption	51(4.7%)	74(6.3%)	0.105	112(5.4%)	13(7.6%)	0.209
Physical Activity (MET-min/week)			< 0.001			0.076
No/Low PA (< 960)	343(32%)	474(40%)		745(36%)	72(42%)	
Medium PA (961–1800)	117(11%)	101(8.6%)		198(9.5%)	20(12%)	
High PA (> 1800)	621(57%)	604(51%)		1147(55%)	1147(55%)	
Hepatitis B/ Hepatitis C	11(1.0%)	26(2.2%)	0.026	31(1.5%)	6(3.5%)	0.055
Family income to poverty ratio	2.03 [1.02,4.17]	2.09 [1.18,3.82]	0.525	2.07 [1.10,3.96]	2.17 [1.23,4.45]	0.230
Age (years)	33.00 [24.00,44.00]	43.00 [32.00,52.00]	< 0.001	38.00 [27.00,48.00]	47.00 [36.00,54.00]	< 0.001
HDL (mmol/L)	1.42 [1.22,1.71]	1.19 [1.01,1.42]	< 0.001	1.29 [1.09,1.58]	1.16 [0.98,1.34]	< 0.001
ALB (g/L)	42.00 [40.00,44.00]	41.00 [39.00,43.00]	< 0.001	41.00 [39.00,44.00]	41.00 [38.00,43.00]	< 0.001
GlB (g/L)	30.00 [28.00,33.00]	31.00 [29.00,34.00]	< 0.001	31.00 [28.00,33.00]	31.00 [29.00,35.00]	0.001
ALT (U/L)	15.00 [12.00,21.00]	22.00 [15.00,33.00]	< 0.001	18.00 [13.00,26.00]	30.00 [18.25,47.00]	< 0.001
AST (U/L)	18.00 [15.00,22.00]	20.00 [16.00,25.00]	< 0.001	19.00 [16.00,23.00]	24.00 [18.00,35.00]	< 0.001
LDH (IU/L)	146.00 [130.00,162.00]	153.00 [138.00,173.00]	< 0.001	149.00 [133.00,168.00]	159.00 [141.00,182.00]	< 0.001
TBIL (μmol/L)	6.84 [5.13,10.26]	6.84 [5.13,8.55]	< 0.001	6.84 [5.13,8.55]	6.84 [5.13,10.26]	0.738
TG (mmol/L)	0.97 [0.72,1.42]	1.56 [1.10,2.29]	< 0.001	1.22 [0.84,1.82]	1.71 [1.12,2.47]	< 0.001

HDL-C, direct HDL-Cholesterol; ALB, albumin; GLB, globulin; ALT, alanine aminotransferase; AST, aspartate aminotransferase; LDH, lactate dehydrogenase; TIBL, Total Bilirubin; TG, triglyceride.

*n (%); Median [25%,75%].

^#^Pearson's *Chi*-squared test; Wilcoxon rank sum test.

### ROC analysis of indicators in the identification of hepatic steatosis/fibrosis patients

An individual is classified as overweight if their BMI is ≥ 25 kg/m^2^ according to the World Health Organization (WHO). However, there is currently no universally accepted standard or definitive cut-off criterion for assessing factors such as the FMI1, FMI2, WWI, BF%, WHR, and ASMI. Given the binary classification needs, we employed ROC analysis to ascertain the optimal threshold FMI1, FMI2, WWI, BF%, WHR, and ASMI values. Tables S1 and S2 demonstrate the optimal cut-off values of the indicators identified by maximizing Youden’s index in the identification of hepatic steatosis and fibrosis patients.

The continuous variables of the seven body composition indices were transformed into dichotomous variables using cut-off values in this research. Table 2 displays the values for the areas under the curve (AUCs), as well as the sensitivity and specificity of the seven categorical anthropometric indicators for identifying hepatic steatosis and fibrosis patients. As shown in Table 2, the WHR and FMI1 achieved the highest AUCs in identifying hepatic steatosis and fibrosis patients, respectively (0.720 and 0.726), which demonstrates the optimal anthropometric indicators among the parameters.

**Table 2:** Performance of the seven anthropometric indicators in the identification of hepatic steatosis and fibrosis patients.

Outcome	Indicator	Sensitivity	Specificity	AUC	95%CI	P value
Steatosis	FMI1	0.845	0.547	0.696	0.678–0.714	< 0.001
	FMI2	0.801	0.562	0.681	0.662–0.700	< 0.001
	WWI	0.798	0.627	0.713	0.694–0.731	< 0.001
	BMI	0.885	0.536	0.711	0.693–0.728	< 0.001
	BF%	0.705	0.519	0.612	0.592–0.632	< 0.001
	**WHR**	**0.735**	**0.706**	**0.720**	**0.702–0.739**	**< 0.001**
	ASMI	0.649	0.668	0.709	0.688–0.730	< 0.001
Fibrosis	**FMI1**	**0.617**	**0.751**	**0.726**	**0.711–0.741**	**< 0.001**
	FMI2	0.659	0.690	0.715	0.700–0.731	< 0.001
	WWI	0.753	0.610	0.717	0.712–0.742	< 0.001
	BMI	0.897	0.333	0.615	0.603–0.627	< 0.001
	BF%	0.734	0.471	0.630	0.613–0.647	< 0.001
	WHR	0.625	0.695	0.715	0.700–0.730	< 0.001
	ASMI	0.800	0.538	0.717	0.702–0.732	< 0.001

FMI1, fat mass index ([fat mass]/height^2^); FMI2, fat mass index ([fat mass]/height^3^); WWI, weight-adjusted-waist index; BMI, body mass index; BF%, percentage of body fat; WHR, waist-to-hip ratio; ASMI, appendicular skeletal muscle index.

Significant values are in bold.

### Association among the seven anthropometric indicators, hepatic steatosis, and fibrosis

As shown in Table 3, higher FMI1, FMI2, WWI, BMI, BF%, WHR, and ASMI values were associated with a higher risk of hepatic steatosis and fibrosis (unadjusted *p* < 0.001). After adjustment for age, sex, race, hypertension, diabetes, education level, smoking, alcohol consumption, the family income to poverty ratio, physical activity, hepatitis B or C and HDL, ALB, GLB, ALT, AST, LDH, TBIL, and TG values, the FMI1 value had the significantly highest OR for both hepatic steatosis (6.40 [4.91–8.38]) and fibrosis (6.06 [5.00, 7.37]) among the seven indicators (Table 3). In Tables S3 and S4., we conducted a detailed stratification of steatosis and fibrosis severity using FibroScan values. The FMI demonstrated the highest OR across various stages and was notably significant in subsets of mild steatosis (≤ S1), advanced steatosis (S2-S3), as well as across the fibrosis spectrum, including significant and severe fibrosis (F2-F3), and cirrhosis (F4).

**Table 3:** Association among the seven anthropometric indicators, hepatic steatosis, and fibrosis.

Outcome	Indicator (cut-off value)	Model1		Model2		Model3		Model4	
Hepatic Steatosis	**FMI1 (7.61)**	**6.56 (5.40–8.02)**	**< 0.001**	**9.44 (7.45–12.05)**	**< 0.001**	**8.92 (7.01–11.43)**	**< 0.001**	**6.40 (4.91–8.38)**	**< 0.001**
	FMI2 **(**4.69**)**	5.14 (4.27–6.21)	< 0.001	8.52 (6.72–10.88)	< 0.001	7.78 (6.11–9.97)	< 0.001	5.87 (4.45–7.81)	< 0.001
	WWI **(**10.62**)**	6.65(5.52–8.04)	< 0.001	6.00 (4.88–7.40)	< 0.001	5.55(4.49–6.88)	< 0.001	3.83 (3.03–4.85)	< 0.001
	BMI **(**25.00**)**	8.92 (7.21–11.10)	< 0.001	8.52 (6.79–10.75)	< 0.001	8.22 (6.52–10.43)	< 0.001	5.37 (4.17–6.97)	< 0.001
	BF% **(**30.65**)**	2.58 (2.17–3.06)	< 0.001	6.92 (5.24–9.24)	< 0.001	6.34 (4.79–8.49)	< 0.001	4.80 (3.54–6.58)	< 0.001
	WHR **(**0.91**)**	6.64 (5.53–7.99)	< 0.001	5.96 (4.84–7.36)	< 0.001	5.46 (4.41–6.79)	< 0.001	3.67 (2.89–4.67)	< 0.001
	ASMI (7.89)	3.72 (3.12–4.43)	< 0.001	6.68 (5.26–8.54)	< 0.001	6.57 (5.15–8.45)	< 0.001	4.43 (3.40–5.81)	< 0.001
Fibrosis	**FMI1 (11.97)**	**4.86 (4.26–5.54)**	**< 0.001**	**7.83 (6.65–9.26)**	**< 0.001**	**7.76 (6.56–9.22)**	**< 0.001**	**6.06 (5.00–7.37)**	**< 0.001**
	FMI2 **(**6.78**)**	4.29 (3.78–4.89)	< 0.001	7.05 (5.99–8.32)	< 0.001	6.86 (5.80–8.14)	< 0.001	5.33 (4.40–6.49)	< 0.001
	WWI **(**10.96**)**	4.77 (4.18–5.44)	< 0.001	4.79 (4.13–5.57)	< 0.001	4.31 (3.70–5.03)	< 0.001	3.04 (2.56–3.61)	< 0.001
	BMI **(**25.00**)**	4.32 (3.66–5.11)	< 0.001	3.61 (3.04–4.30)	< 0.001	3.50 (2.92–4.21)	< 0.001	2.30 (1.86–2.86)	< 0.001
	BF% **(**31.75**)**	2.45 (2.16–2.79)	< 0.001	4.03 (3.42–4.77)	< 0.001	3.91 (3.29–4.66)	< 0.001	2.85 (2.35–3.46)	< 0.001
	WHR **(**0.95**)**	3.61 (3.18–4.10)	< 0.001	2.90 (2.52–3.35)	< 0.001	2.65 (2.29–3.07)	< 0.001	1.67 (1.41–1.98)	< 0.001
	ASMI (7.93)	4.51 (3.94–5.17)	< 0.001	6.37 (5.38–7.56)	< 0.001	6.15 (5.17–7.35)	< 0.001	5.18 (4.25–6.35)	< 0.001

Model 1: Crude model.

Model 2: Adjusted for age, sex, and race.

Model 3: Model 2 in addition to hypertension, diabetes, education level, smoking at least 100 cigarettes in one’s lifetime, alcohol consumption, and the family income to poverty ratio.

Model 4: Model 3 in addition to physical activity, hepatitis B or C, HDL cholesterol, albumin, globulin, ALT, AST, LDH, total bilirubin, physical activity and triglyceride levels.

Abbreviations: FMI1, fat mass index ([fat mass]/height^2^); FMI2, fat mass index ([fat mass]/height^3^); WWI, weight-adjusted-waist index; BMI, body mass index; BF%, percentage of body fat; WHR, waist-to-hip ratio; ASMI, appendicular skeletal muscle index.

Significant values are in bold.

### Subgroup analysis

To clarify the stability of the outcomes obtained from multivariate regression analysis, we conducted a stratified multivariate regression analysis to study the relationship between the FMI1 value and the likelihood of developing hepatic steatosis and fibrosis in various subgroups within the population. The analysis was stratified based on demographic factors, including age, sex, race, education level, the family income to poverty ratio, and health-related factors, such as smoking, physical activity, hepatitis B or C, hypertension, and diabetes.

**Figure 1:**
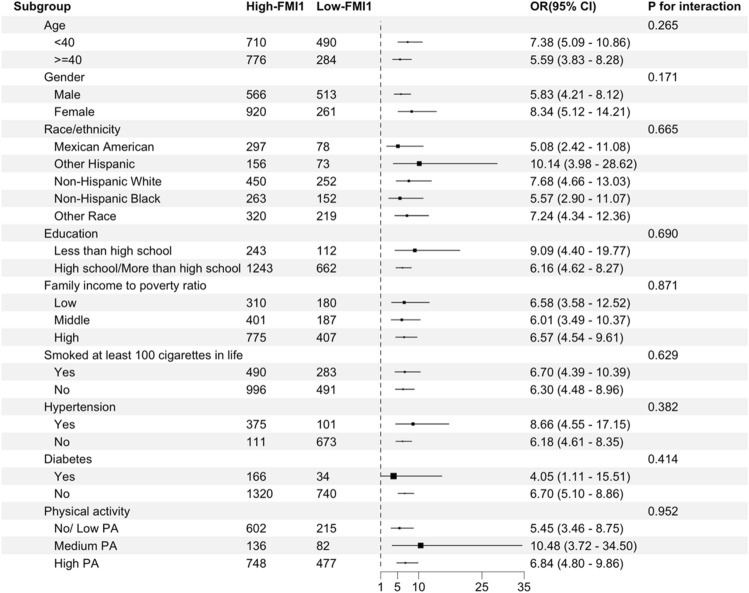
Stratified associations between the FMI1 value and hepatic steatosis. All ORs were calculated by adjusting for age, sex, race, hypertension, diabetes, education level, smoking at least 100 cigarettes in one’s lifetime, alcohol consumption, the family income to poverty ratio, physical activity, hepatitis B or C, HDL cholesterol, albumin, globulin, ALT, AST, LDH, total bilirubin, and triglyceride levels.

**Figure 2:**
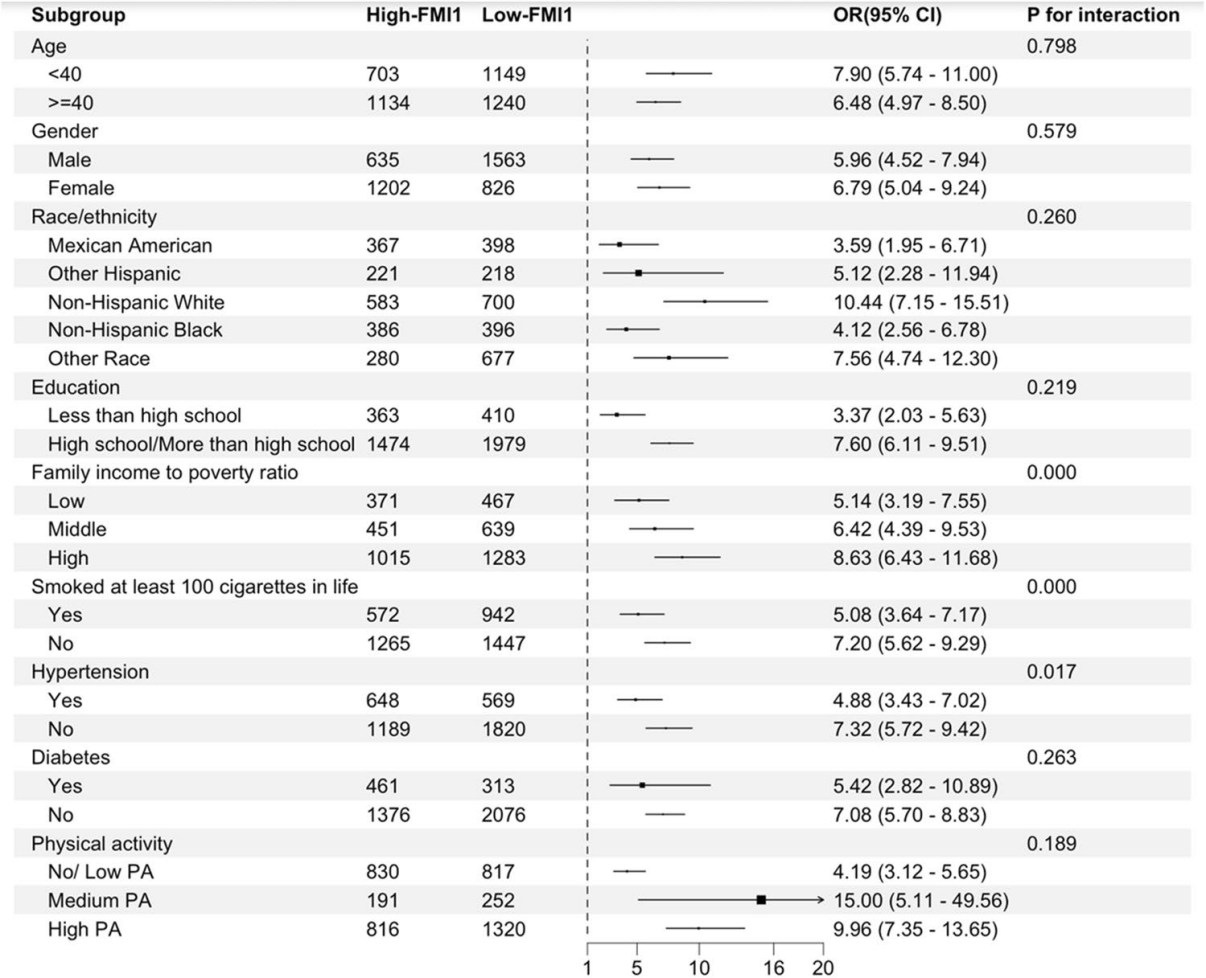
Stratified associations between the FMI1 value and liver fibrosis. All ORs were calculated by adjusting for age, sex, race, hypertension, diabetes, education level, smoking at least 100 cigarettes in one’s lifetime, alcohol consumption, the family income to poverty ratio, physical activity, hepatitis B or C, HDL cholesterol, albumin, globulin, ALT, AST, LDH, total bilirubin, and triglyceride levels.

As illustrated in Fig. 1, the risk of developing hepatic steatosis among individuals with high FMI1 values was consistent in the abovementioned subgroups. Additionally, Fig. 2 reveals that among individuals with a high family income to poverty ratio, individuals with no smoking history, and nonhypertensive individuals, those with high FMI1 values exhibited increased susceptibility to liver fibrosis. Significant disparities were identified in the interaction test. There may be potential interaction effects between the family income to poverty ratio, smoking, hypertension, and FMI1 value that are associated with the presence of liver fibrosis (*p* for interaction < 0.05).

To investigate the impact of seven anthropometric indicators on nonobese steatosis, multivariate analyses were carried out among nonobese participants (n = 1546) (Table S5.) The FMI1 had the significantly highest OR for nonobese steatosis (4.80 [2.70–8.80]) among the seven indicators in Model 4.

### The association of the FMI1 value with the CAP and LSM values

**Figure 3:**
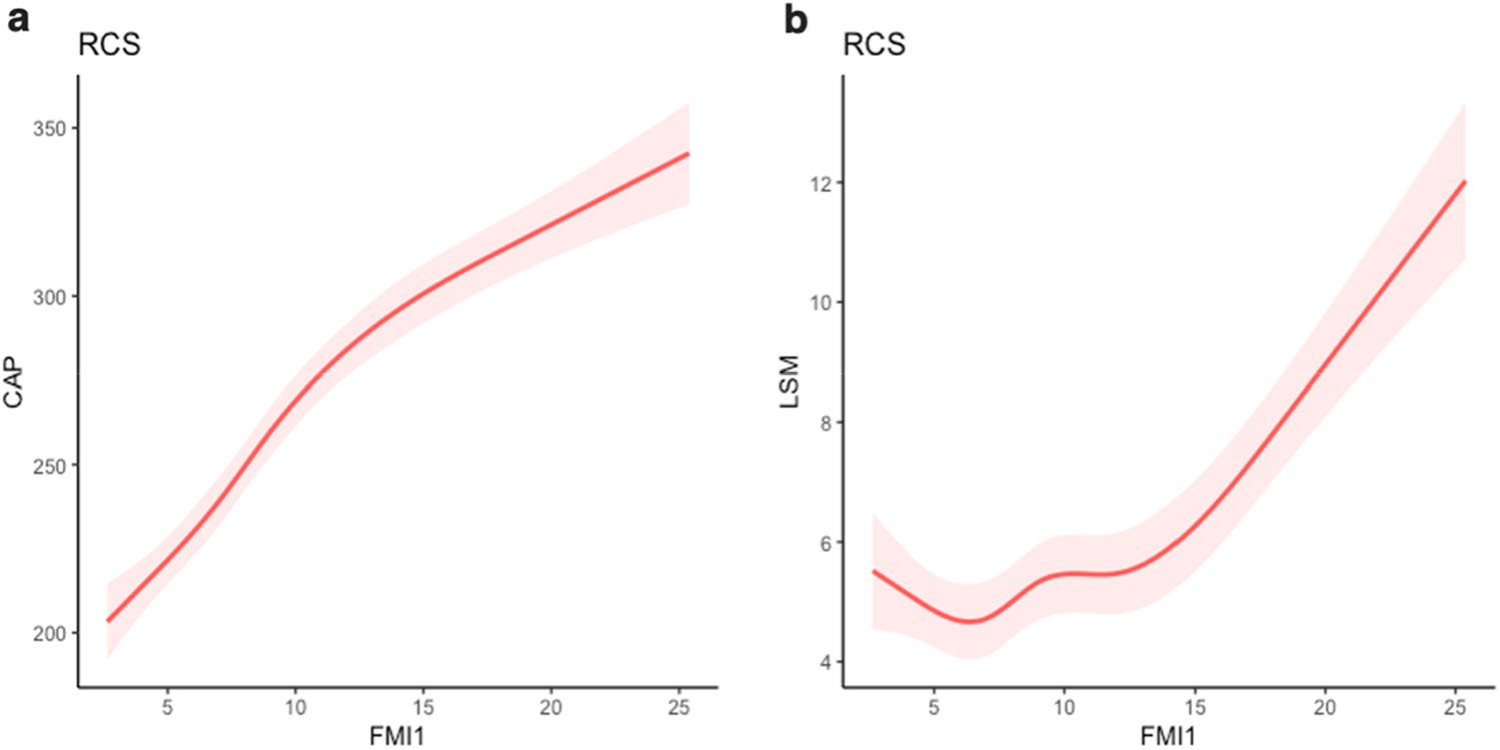
Nonlinear association of the FMI1 value with the CAP (**a**) and LSM (**b**) values according to the restricted cubic spline model adjusted for age, sex, race, hypertension, diabetes, education level, smoking at least 100 cigarettes in one’s lifetime, alcohol consumption, the family income to poverty ratio, physical activity, hepatitis B or C, HDL cholesterol, albumin, globulin, ALT, AST, LDH, total bilirubin, and triglyceride levels. The red dashed line represents the upper and lower bounds of the 95% confidence intervals.

Both the CAP and LSM values showed a nonlinear connection with the FMI1 value (*p* for nonlinearity < 0.001). In Fig. 3, restricted cubic spline (RCS) models were employed to flexibly model and visually represent the nonlinear relationship between the FMI1 value and the CAP and LSM values after adjusting for all variables in Model 4. The RCS models show a significant positive correlation of the FMI1 value with the CAP and LSM values (Fig. 3).

### Sharp regression discontinuity plot of the FMI1 value versus hepatic steatosis and fibrosis

**Figure 4:**
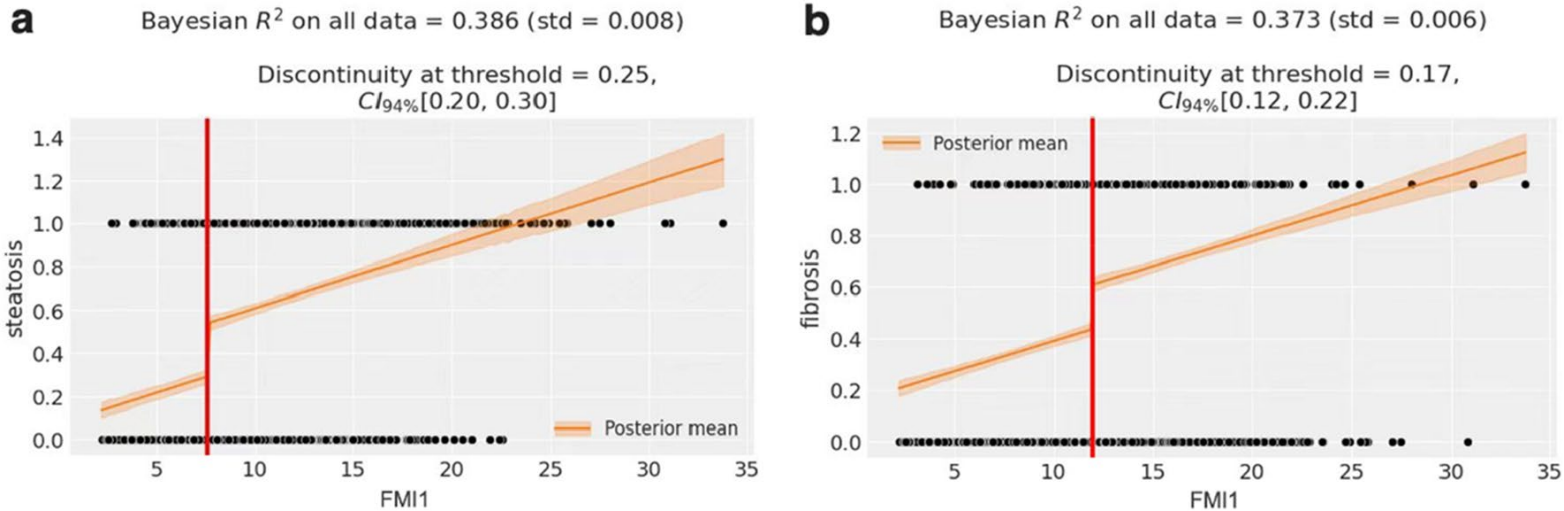
The graphical depiction of the effect observed at the threshold in hepatic steatosis (**a**) and fibrosis (**b**). The ‘jump’ observed in the regression line at the threshold signifies the impact of the FMI1 cut-off value on the diagnosis of hepatic steatosis and fibrosis and the intention-to-treat effect at the threshold.

Figure 4 shows a noticeable increase in hepatic steatosis at the FMI1 cut-off value of 7.61, with noticeable discontinuities. Similarly, there was a discontinuous decrease in liver fibrosis at the FMI1 threshold value of 11.97. The ‘jump’ observed in the regression line at the threshold signifies the impact of the FMI1 cut-off value on the diagnosis of hepatic steatosis and fibrosis and the intention-to-treat effect at the threshold.

## Discussion

This study evaluated the correlation between seven anthropometric indicators and the presence of hepatic steatosis and fibrosis. Given the cross-sectional population-based design, we observed a significant correlation between the FMI value and the presence of hepatic steatosis and fibrosis.

Obesity is well established to be linked to lipid metabolism disorders, which not only play a role in the occurrence and progression of hepatic steatosis but also pose a risk of metabolic syndromes^11^. BMI is extensively employed as a means of assessing the degree of obesity. While NAFLD is commonly observed in individuals who are obese or have diabetes mellitus, up to 7% to 20% of cases of NAFLD are diagnosed in individuals with a BMI < 25 kg/m^2^^12^. Recent research indicates that individuals diagnosed with lean NAFLD have a similar or higher incidence of liver fibrosis than people with nonlean NAFLD^13^. Body weight measurement is not a precise substitute for assessing total body fat mass by anthropometric means^14^. Additionally, the discriminative ability of BMI is restricted in regard to differentiating between muscle and adipose tissue components^9^. This suggests that BMI may not be the most reliable anthropometric indicator for screening hepatic steatosis and fibrosis.

The key factor driving the development of nonalcoholic steatohepatitis, insulin resistance, and cardiovascular disease has been identified as central obesity, more precisely the accumulation of adipose tissue in deep subcutaneous tissue^15^. Moreover, previous research has indicated that adipose tissue distribution, including abdominal fat distribution, is increasingly related to hepatic steatosis and metabolic syndrome^16–18^. The WHR can serve as a reliable clinical marker of the distribution of abdominal fat and central obesity^19^. In this context, we found that the WHR showed a more remarkable association with hepatic steatosis than BMI.

Recent studies found that an increased fat mass is linked to increased hepatic steatosis risk^20,21^. Furthermore, another analysis revealed that adipose tissue accumulation is a significant contributing factor in the development of NAFLD among male Japanese young adults, even among nonobese persons^22^. In our research, multivariable logistic analysis indicated that high-fat accumulation assessed by higher FMI values showed positively and independently association with the development of hepatic steatosis. The findings were further confirmed by a study of physical examination data from 56,639 Japanese participants that evaluated subjects with FMI-predominant and fat-free mass index-dominant body compositions and showed that there was a significant independent association between the predominance of fat mass in qualitative body composition changes and the presence of fatty liver in both men and women^23^. According to Burton, the utilization of the dimensionless quantity (fat mass)/height^3^ is a more appropriate method for determining the FMI in children and adolescents compared to the use of (fat mass)/height^2^ due to their closer resemblance to BF% curves^24^. However, BF% fails to account for the distinct contributions of fat and lean body mass, which could lead to the inability to recognize individuals with high fat and lean body mass^25^. Furthermore, the connection between height and (fat mass)/height^3^ was found to be lower than the correlation between height and (fat mass)/height^2^. The question of whether the best indicator of adiposity should be independent of height remains ambiguous because this may obscure biological relationships between adiposity and height^26^.

An estimate of the degree of liver fibrosis can be assessed by the LSM value, while hepatic steatosis can be quantified by using the CAP value^27,28^. For this study, we found a significant positive correlation among FMI, CAP, and LSM values. The connection among fat accumulation, hepatic steatosis, and fibrosis has several explanations. The overflow hypothesis claims that subcutaneous tissue has a limited capacity to increase the size and quantity of adipocytes and that each person’s ability to store lipids in adipose tissues is restricted^29,30^. Under such conditions, lipids tend to mainly concentrate in the liver tissue, resulting in the development of hepatic steatosis^29,30^. Adipose tissue has the potential to continuously release proinflammatory stimuli, hence contributing to the development of systemic inflammation, metabolic problems, and histological changes in the liver^31^. In addition, a proposal revealed that an imbalance in lipid metabolism contributes to the creation of lipotoxic intermediates, which in turn lead to oxidative stress and endoplasmic reticulum stress, trigger inflammasome activation, and initiate apoptotic cell death, followed by inflammation, tissue regeneration, and fibrogenesis^32^.

We reported that the FMI is an independent risk factor for the development of liver fibrosis. The FMI has been identified as an effective tool for the identification of patients with metabolic syndrome^33,34^, which is a cause and a consequence of NAFLD^35^. Certain subtypes of ceramidases produced from adipose tissue have the potential to induce insulin desensitization and hence contribute to the development of insulin resistance^36^. Insulin resistance leads to the advancement of fibrosis in nonalcoholic steatohepatitis through mechanisms such as hepatocyte cell death, the increased generation of reactive oxygen species, and disturbances in the balance of adipokines and cytokines^37^. The presence of liver fibrosis in individuals with fatty liver is commonly linked to metabolic disorders, including diabetes mellitus and dyslipidaemia, rather than BMI^37^. In our study, the FMI exhibited superior efficacy as a screening tool for identifying the presence of liver fibrosis compared with the WWI, BMI, BF%, WHR, and ASMI. We further validated the effect of the FMI threshold on the diagnosis of hepatic steatosis and fibrosis through sharp regression discontinuity.

Based on subgroup analysis, we observed that the family income to poverty ratio, smoking, and hypertension affected the association between the FMI value and liver fibrosis risk. The interaction can be further investigated and verified by RCT studies with a larger population in the future. Moreover, the mechanism of the interaction remains to be determined and could be investigated in the future through basic experiments.

This study had some strengths. We employed an oversampling technique to address the imbalance in hepatic fibrosis sample sizes. We conducted a comparative analysis to assess the ability of seven anthropometric indicators to identify hepatic steatosis and fibrosis patients, and we found a strong association between the FMI value and both hepatic steatosis and fibrosis. Moreover, to account for potential confounding variables, we distinguished the likely protected population with higher FMI values by stratified analysis. Finally, the rationale and validity of the cut-off values of several anthropometric indicators for identifying hepatic steatosis and fibrosis were further confirmed using SRD analysis.

Nonetheless, several limitations to this study should be mentioned. First, we are unable to draw any conclusions on the cause-and-effect relationship between the FMI value and hepatic steatosis or fibrosis due to the limitations of the cross-sectional study design. Second, VCTE was used to diagnose hepatic steatosis and fibrosis in this investigation; however, additional validation is required in a population of patients with confirmed biopsy results. Third, it should be noted that while the NHANES was carried out in the United States and included a multi-ethnic adult population, the generalizability of the findings to other geographical regions or ethnicities requires further validation. Then, it is necessary to conduct a larger cohort to further investigate the associations between the FMI and different stages of liver disease, specifically mild or advanced steatosis and mild (F1), significant and severe fibrosis (F2–F3), and cirrhosis (F4) in the future. Last, given that it is unclear how the FMI and each subgroup interact with each other in patients with liver fibrosis, future research should be conducted to further investigate the precise mechanism underlying our findings.

## Materials and methods

### Data and sample sources

The NHANES, which is conducted biennially by the National Center for Health Statistics (NCHS), is a comprehensive study designed to obtain information about the nutritional and physical health status of the general population in the United States through interviews, physical examinations, and laboratory tests. The survey received approval from the Centers for Disease Control and Prevention Research Ethics Review Board, and the informed permission of all participants was documented.

**Figure 5:**
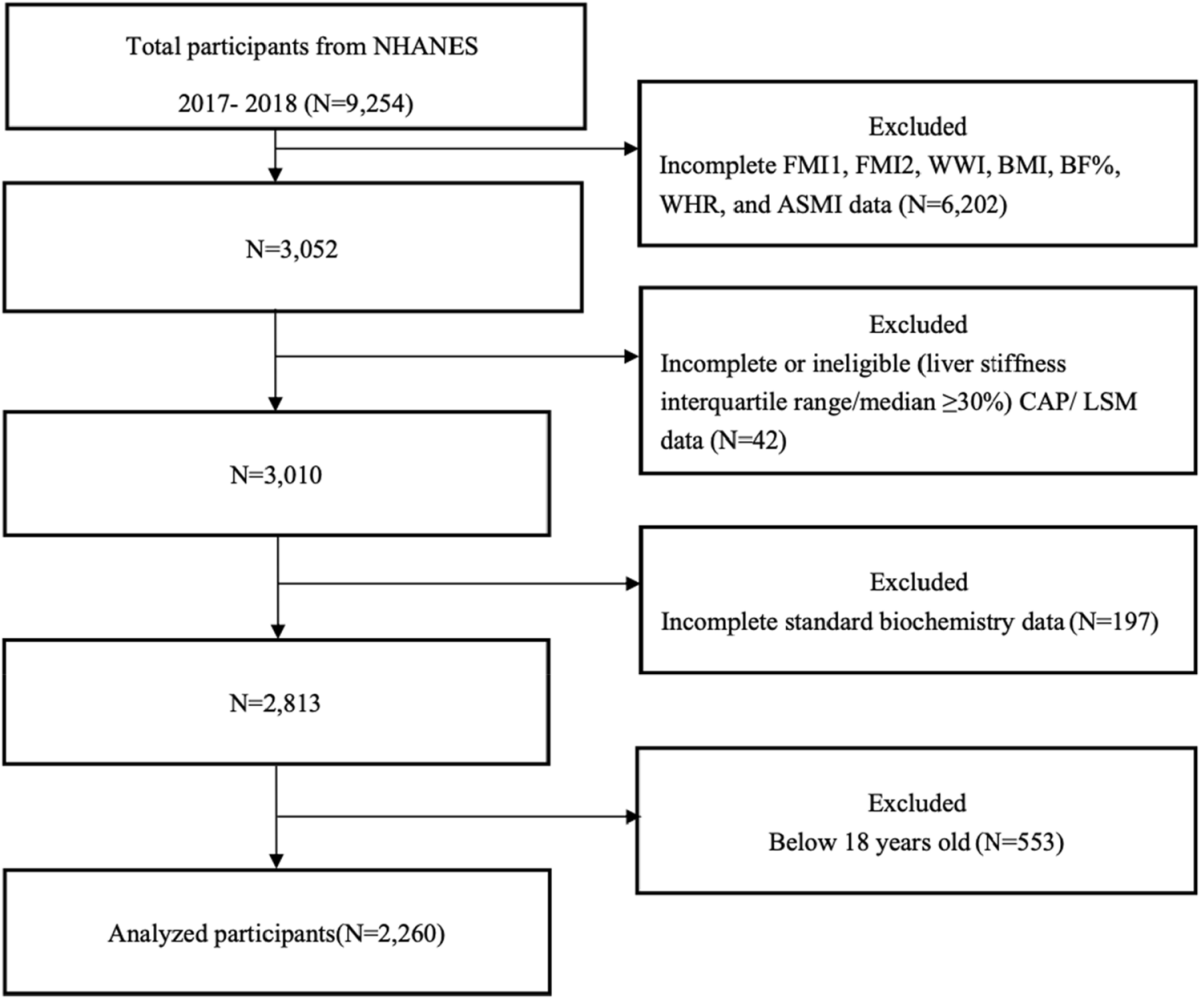
Flowchart of the inclusion of study participants. Abbreviations: NHANES, National Health and Nutrition Examination Survey; FMI1, fat mass index ([fat mass]/height^2^); FMI2, fat mass index ([fat mass]/height^3^); WWI, weight-adjusted-waist index; BMI, body mass index; BF%, percentage of body fat; WHR, waist-to-hip ratio; ASMI, appendicular skeletal muscle index; CAP, controlled attenuation parameter; LSM, liver stiffness measurement.

The present research utilized 2017–2018 data obtained from the NHANES database. Among the total sample of 9254 participants, we excluded those with incomplete FMI1 ([fat mass]/height^2^), FMI2 ([fat mass]/height^3^), WWI, BMI, BF%, WHR, and ASMI data (n = 6202), those with incomplete or ineligible (a liver stiffness interquartile range/median value ≥ 30%) controlled attenuation parameter (CAP)/liver stiffness measurement (LSM) data (n = 42), and those without standard biochemistry data (n = 197). Additionally, individuals who were younger than 18 years old (n = 553) were also excluded from the analysis. The final analysis was conducted with a sample size of 2260 (Fig. 5).

### Definition and measurement of the seven anthropometric indicators

The fat mass, appendicular skeletal muscle mass and BF% values were measured by DXA. The waist circumference, hip circumference, weight, and height measurements were conducted within a mobile examination center, where tests were performed in controlled conditions. Muscle mass was quantified through the employment of the ASMI which is a standardized measure. The FMI1, FMI2, WWI, BMI, BF%, WHR, and ASMI were incorporated as exposure variables in our research, and the computation was performed in the following manner:

1 $$\begin{array}{c}{\text{FMI}}{1} = \text{fat mass(kg)}/{\text{height(m)}}^{2}\end{array}$$

2 $$\begin{array}{c}{\text{FMI}}{2} = \text{fat mass(kg)}/{\text{height(m)}}^{3}\end{array}$$

3 $$\begin{array}{c}{\text{WWI}} = \text{waist circumference(cm)}/\sqrt{\text{weight(kg)}}\end{array}$$

4 $$\begin{array}{c}{\text{BMI}} = \text{weight(kg)}/{\text{height(m)}}^{2}\end{array}$$

5 $$\begin{array}{c}{\text{WHR}} = \text{waist circumference(cm)}/\text{hip circumference(cm)}\end{array}$$

6 $$\begin{array}{c}{\text{ASMI}} = \text{appendicular skeletal muscle mass (kg)}/{\text{height(m)}}^{2}\end{array}$$

### Assessment of hepatic steatosis and fibrosis

The assessment of liver fibrosis was carried out by FibroScan®, which employs ultrasound and VCTE to record the liver stiffness measurement (LSM) value. The instrument concurrently calculates the ultrasonic attenuation of the echo wave linked to hepatic steatosis and derives the controlled attenuation parameter (CAP) as an index for hepatic steatosis^38^. The elastography measurements were captured using the FibroScan model 502 V2 Touch, which was equipped with either a medium (M) or extra-large (XL) probe, as documented in the NHANES database. Individuals were classified as having hepatic steatosis (≤ S1) if the CAP value was ≥ 248 dB/m^39^. A median LSM value ≥ 8 kPa was regarded as an indication of mild (≥ F1) liver fibrosis^40^.

### Covariates

The selection of covariates was informed by previous research. Demographic data comprised sex, age, race, educational level, and the family income to poverty ratio. Additionally, our study included personal life history data obtained from self-report questionnaires regarding smoking status (whether they smoked at least 100 cigarettes in their lifetime), alcohol status (whether they drank an average of ≥ 20 g/day and ≥ 30 g/day for women and men, respectively), physical activity status, hepatitis status (whether they had hepatitis B or hepatitis C), hypertension history, and diabetes history. The variables encompassed in the laboratory data were direct high-density lipoprotein cholesterol (HDL, mmol/L), albumin (ALB, g/L), globulin (GLB, g/L), alanine aminotransferase (ALT, U/L), aspartate aminotransferase (AST, U/L), lactate dehydrogenase (LDH, IU/L), total bilirubin (TBIL, µmol/L), and triglyceride (TG, mmol/L) levels. More thorough details of all variables can be acquired from the NHANES database website (https://www.cdc.gov/nchs/nhanes/index.htm).

### Statistical analysis

We imputed and interpolated missing covariate values with the random forest algorithm using the mice (version 3.16.0) package in the R program (version 4.3.1)^41^. We used the oversampling method in the ROSE (version 0.0.4) package to address liver fibrosis sample imbalance. The median and interquartile ranges are used to report quantitative variables. Qualitative data are expressed in terms of patient counts and percentages (%). The Wilcoxon rank test was used to evaluate differences across several groups for continuous data, while the *chi*-squared test was utilized for categorical variables.

The optimal cut-off values of different indices were selected by maximizing Youden’s index. The continuous variables of the seven body composition indices were transformed into dichotomous variables using cut-off values in the following research. Using receiver operating characteristic curves, the discriminative ability of each body composition categorical variable was determined by calculating the area under the curve (AUC) with a 95% confidence interval (CI). To further investigate the relationship between the FMI1, FMI2, WWI, BMI, WHR, ASMI, and BF% values and hepatic steatosis and fibrosis, we constructed four types of separate multivariable logistic regression models that adjusted for different variables (Models I–IV). The odds ratios (ORs) and 95% CIs of the results are presented. Furthermore, stratified analysis was conducted to identify the stratified connection between the FMI1 value and hepatic steatosis as well as fibrosis.

Using restricted cubic splines with five knots at the 5th, 27.5th, 50th, 72.5th, and 95th percentage points, the relationship among the FMI, CAP, and LSM values was investigated. A sharp regression discontinuity (SRD) analysis was used to explore the impact of FMI1 cut-off values on the diagnosis of hepatic steatosis and fibrosis. All studies were carried out using R version 4.3.1 and Python version 3.11.3. A two-sided *p* value less than 0.05 was indicative of statistical significance in our study.

### Institutional review board statement

NHANES was conducted in accordance with the Declaration of Helsinki, and approved by the NCHS Research Ethics Review Board (Continuation of Protocol #2011–17 and Protocol #2018–01, 26 October 2017). We used publicly available datasets from the official NHANES website (https://wwwn.cdc.gov/nchs/nhanes). Therefore, the need for ethical approval was not applicable.

## Conclusions

This study found a strong association of the FMI value with hepatic steatosis and fibrosis and suggested that the FMI value may be a potential anthropometric indicator to identify patients with liver diseases.

### Supplementary Information

Supplementary Information.

## Data Availability

All NHANES data for this study are publicly available and can be found here: https://wwwn.cdc.gov/nchs/nhanes.
