# Supplementary material for: Association of the fat mass index with hepatic steatosis and fibrosis: evidence from NHANES 2017–2018
Source: Sci Rep. 2024 Mar 23;14:6943. doi: 10.1038/s41598-024-57388-1 (PMC10960854; doi:10.1038/s41598-024-57388-1)
Supplement: Supplementary file 1 — Supplementary Information. [file 41598_2024_57388_MOESM1_ESM.pdf]

Supporting information

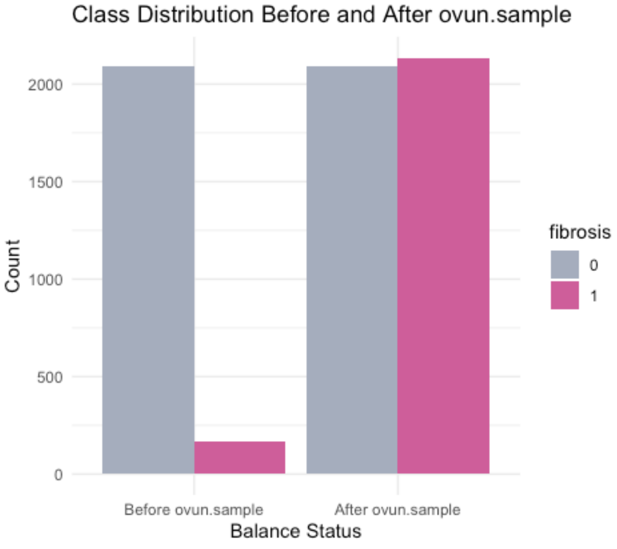

Figure S1. Class distribution before and after oversampling in liver fibrosis sample.

Steatosis classification was based on the percentage of affected hepatocytes: healthy (S0, < 5 or 10% depending on the trial), mild (≤ S1, 5 or 10-33%), advanced steatosis (S2-S3, > 34%). Individuals were classified as having mild steatosis or advanced steatosis if the CAP value was 248-268 dB/m or ≥ 268 dB/m, respectively[39]. A median LSM value of 8-8.5, 8.5-14.6, and ≥ 14.6 kPa were regarded as an indication of mild fibrosis (≥F1), significant and severe fibrosis (F2-F3), and cirrhosis (F4)[40]. The severity of steatosis and fibrosis was further stratified according to FibroScan values (mild steatosis: 207 vs. advanced steatosis: 972; mild fibrosis: 37 vs. significant and severe fibrosis: 95 vs. cirrhosis: 38). Table S1 and Table S2 demonstrate the optimal cut-off values of the indicators identified by maximizing Youden's index in the identification of hepatic steatosis or advanced steatosis and mild fibrosis, significant fibrosis, severe fibrosis, or cirrhosis.

Table S1. The optimal cut-off values of the indicators were identified by maximizing Youden's index in the identification of steatosis or advanced steatosis.

Indicators	Steatosis	Mild steatosis (≤ S1)	Advanced steatosis (S2-S3)
FMI1	7.61	7.55	8.50
FMI2	4.69	4.40	4.70
WWI	10.62	10.62	10.60
BF%	30.65	29.15	26.25
WHR	0.91	0.90	0.91
ASMI	7.89	6.79	7.88

Abbreviations: FMI1, fat mass index ([fat mass]/height^2); FMI2, fat mass index ([fat mass]/height^3); WWI, weight-adjusted-waist index; BF%, percentage of body fat; WHR, Waist-to-Hip Ratio; ASMI, appendicular skeletal muscle index.

.

Table S2. The optimal cut-off values of the indicators were identified by maximizing Youden's index in the identification of mild fibrosis, significant fibrosis, severe fibrosis, or cirrhosis.

Indicators	Fibrosis	Mild fibrosis (≥F1)	Significant and severe fibrosis (F2-F3)	Cirrhosis (F4)
FMI1	11.97	12.28	11.97	10.17
FMI2	6.78	8.38	6.78	5.69
WWI	10.96	11.35	11.01	10.97
BF%	31.75	40.65	29.75	32.35

WHR	0.95	0.92	0.96	0.95
ASMI	7.93	8.40	8.51	7.72

Abbreviations: FMI1, fat mass index ([fat mass]/height^2); FMI2, fat mass index ([fat mass]/height^3); WWI, weight-adjusted-waist index; BF%, percentage of body fat; WHR, Waist-to-Hip Ratio; ASMI, appendicular skeletal muscle index.

The continuous variables of the seven body composition indices were transformed into dichotomous variables using cut-off values in the following research. We constructed four types of separate multivariable logistic regression models that adjusted for different variables (Models I-IV). The odds ratios (ORs) and 95% CIs of the results are presented in Table S3. and Table S4.

Table S3. Association among the seven anthropometric indicators, mild steatosis, and advanced steatosis.

Outcome	Indicator (cut-off value)	Model1		Model2		Model3		Model4	
Mild Steatosis (≤ S1)	FMI1 (7.55)	4.25 (3.01-6.09)	<0.001	4.57 (3.13-6.79)	<0.001	4.43 (3.01-6.63)	<0.001	3.87 (2.58-5.92)	<0.001
	**FMI2 (4.40)**	**4.12 (2.89-5.98)**	**<0.001**	**4.65 (3.12-7.05)**	**<0.001**	**4.48 (2.99-6.85)**	**<0.001**	**3.96 (2.58-6.17)**	**<0.001**
	WWI (10.62)	4.46 (3.23-6.24)	<0.001	3.59 (2.54-5.12)	<0.001	3.31 (2.31-4.77)	<0.001	2.81(1.92-4.16)	<0.001
	BMI (25.00)	3.93 (2.80-5.60)	<0.001	3.75 (2.63-5.42)	<0.001	3.60 (2.52-5.22)	<0.001	2.89 (1.97-4.31)	<0.001
	BF% (29.15)	2.66 (1.90-3.78)	<0.001	3.50 (2.26-5.47)	<0.001	3.30 (2.12-5.19)	<0.001	2.89(1.82-4.64)	<0.001
	WHR (0.90)	4.21 (3.07-5.81)	<0.001	4.34 (3.07-6.20)	<0.001	3.97 (2.77-5.73)	<0.001	3.28 (2.25-4.82)	<0.001
	ASMI (6.79)	2.01 (1.45-2.83)	<0.001	3.77 (2.54-5.67)	<0.001	3.60 (2.41-5.44)	<0.001	2.86 (1.87-4.41)	<0.001
Advanced steatosis (S2-S3)	**FMI1 (8.50)**	**4.99 (4.14-6.04)**	**<0.001**	**8.56 (6.76-10.91)**	**<0.001**	**8.00(6.30-10.23)**	**<0.001**	**5.81 (4.47-7.62)**	**<0.001**
	FMI2 (4.70)	4.99 (4.10-6.11)	<0.001	8.89 (6.95-11.46)	<0.001	8.24 (6.42-10.66)	<0.001	5.80 (4.41-7.67)	<0.001
	WWI (10.60)	5.93(4.87-7.26)	<0.001	5.72(4.60-7.16)	<0.001	5.38 (4.30-6.77)	<0.001	3.66 (2.86-4.70)	<0.001
	BMI (25.00)	9.23(7.25-11.88)	<0.001	8.54 (6.63-11.10)	<0.001	8.20 (6.34-10.71)	<0.001	5.31 (4.01-7.09)	<0.001
	BF% (26.25)	3.73 (2.95-4.75)	<0.001	5.99 (4.52-7.99)	<0.001	3.91 (3.29-4.66)	<0.001	4.20 (3.08-5.77)	<0.001
	WHR (0.91)	5.79 (4.81-6.99)	<0.001	2.90 (2.52-3.35)	<0.001	4.37 (3.53-5.42)	<0.001	2.68 (2.12-3.39)	<0.001
	ASMI (7.88)	4.18 (3.5-5.01)	<0.001	6.83 (5.37-8.75)	<0.001	6.64 (5.20-8.54)	<0.001	4.56 (3.50-5.98)	<0.001

Model 1: Crude model.

Model 2: Adjusted for age, sex, and race.

Model 3: Model 2 in addition to hypertension, diabetes, education level, smoking at least 100 cigarettes in one's lifetime, alcohol consumption, and the family income to poverty ratio.

Model 4: Model 3 in addition to physical activity, hepatitis B or C, HDL cholesterol, albumin, globulin, ALT, AST, LDH, total bilirubin, and triglyceride levels.

Abbreviations: FMI1, fat mass index ([fat mass]/height^2); FMI2, fat mass index ([fat mass]/height^3); WWI, weight-adjusted-waist index; BMI, body mass index; BF%, percentage of body fat; WHR, waist-to-hip ratio; ASMI, appendicular skeletal muscle index.

Table S4. Association among the seven anthropometric indicators, mild fibrosis, significant and severe fibrosis, and cirrhosis.

Outcome	Indicator (cut-off value)	Model1		Model2		Model3		Model4	
	FMI1 (12.28)	2.56 (1.31-4.93)	0.005	4.77 (3.26-20.51)	<0.001	4.18 (1.83-9.55)	<0.001	4.43 (1.79-11.06)	0.002
	FMI2 (8.38)	3.25 (1.61-6.30)	<0.001	8.13 (3.31-20.33)	<0.001	7.75 (3.06-20.17)	<0.001	8.03 (2.95-22.80)	<0.001
Mild fibrosis (≥F1)	WWI (11.35)	3.12 (1.61-6.01)	<0.001	2.81 (1.38-5.72)	<0.001	2.23 (1.07-4.65)	<0.001	1.85 (0.87-3.98)	0.114
	BMI (25.00)	2.14 (0.99-5.31)	0.072	1.82 (0.82-4.61)	<0.001	1.63 (0.72-4.17)	0.267	1.18 (0.47-3.35)	0.649
	**BF% (40.65)**	**2.46 (1.24-4.74)**	**0.008**	**12.08 (3.58-46.76)**	**<0.001**	**12.24 (3.42-53.29)**	**<0.001**	**13.37(3.53-60.47)**	**<0.001**
	WHR (0.92)	2.76 (1.39-5.84)	0.005	1.82 (0.82-4.18)	0.147	1.42 (0.62-3.39)	0.416	1.07 (0.45-2.63)	0.935
	ASMI (8.40)	1.83 (0.95-3.53)	0.069	1.90 (0.87-4.25)	0.011	1.64 (0.74-3.70)	0.230	1.34 (0.77-3.19)	0.502
	FMI1 (11.97)	5.08 (3.33-7.86)	<0.001	7.68 (4.74-12.60)	<0.001	6.84 (4.16-11.41)	<0.001	7.17 (4.15-12.60)	<0.001
	**FMI2 (6.78)**	**4.53 (2.95-7.11)**	**<0.001**	**7.70 (4.66-12.91)**	**<0.001**	**6.75 (4.04-11.45)**	**<0.001**	**7.19 (4.08-12.93)**	**<0.001**
Significant and severe	WWI (11.01)	3.78 (2.45-5.99)	<0.001	3.41 (2.14-5.55)	<0.001	2.97 (1.84-4.90)	<0.001	2.32 (1.40-3.93)	<0.001
fibrosis (F2-F3)	BMI (25.00)	4.19 (2.27-8.64)	<0.001	3.56 (1.91-7.41)	<0.001	3.20 (1.70-6.69)	<0.001	2.57 (1.27-5.78)	<0.001
	BF% (29.75)	2.92 (1.74-5.21)	<0.001	4.50 (2.54-8.39)	<0.001	3.95 (2.21-7.42)	<0.001	4.10 (2.18-8.13)	<0.001
	WHR (0.96)	3.75 (2.47-5.75)	<0.001	3.27 (2.01-5.35)	<0.001	2.82 (1.71-4.70)	<0.001	2.05 (1.21-3.51)	<0.001
	ASMI (8.51)	4.64 (2.99-7.38)	<0.001	7.17 (4.22-12.48)	<0.001	6.49 (3.79-11.38)	<0.001	6.13 (3.44-1.13)	<0.001
	FMI1 (10.17)	12.58 (4.99-42.25)	<0.001	17.48 (6.61-60.40)	<0.001	15.86 (5.89-55.43)	<0.001	13.12 (4.56-48.65)	<0.001
	**FMI2 (5.69)**	**20.87 (6.36-128.63)**	**<0.001**	**30.30 (8.87-190.03)**	**<0.001**	**27.72 (7.98-175.11)**	**<0.001**	**26.83 (7.04-184.33)**	**<0.001**
Cirrhosis (F4)	WWI (10.97)	26.59 (8.10-163.89)	<0.001	26.80(7.88-167.89)	<0.001	24.68 (7.18-155.39)	<0.001	22.41 (6.12-150.04)	<0.001
	BMI (25.00)	8.49 (2.59-52.33)	0.003	7.35 (2.20-45.62)	<0.001	6.37 (1.89-39.77)	<0.001	5.72 (1.46-42.46)	<0.001
	BF% (32.35)	6.28 (2.67-18.39)	<0.001	12.87 (4.96-39.94)	<0.001	11.59 (4.38-36.56)	<0.001	9.56 (3.41-31.97)	<0.001
	WHR (0.95)	8.43 (3.92-20.94)	<0.001	9.71 (4.18-25.41)	<0.001	9.26 (3.87-24.80)	<0.001	7.29 (2.84-20.86)	<0.001

	ASMI (7.72)	16.33 (4.98-100.66)	<0.001	3.14 (8.85-200.26)	<0.001	26.60 (7.43-170.52)	<0.001	24.11 (6.18-170.46)	<0.001

Model 1: Crude model.

Model 2: Adjusted for age, sex, and race.

Model 3: Model 2 in addition to hypertension, diabetes, education level, smoking at least 100 cigarettes in one’s lifetime, alcohol consumption, and the family income to poverty ratio.

Model 4: Model 3 in addition to physical activity, hepatitis B or C, HDL cholesterol, albumin, globulin, ALT, AST, LDH, total bilirubin, and triglyceride levels.

Abbreviations: FMI1, fat mass index ([fat mass]/height^2); FMI2, fat mass index ([fat mass]/height^3); WWI, weight-adjusted-waist index; BMI, body mass index; BF%, percentage of body fat; WHR, waist-to-hip ratio; ASMI, appendicular skeletal muscle index.

Table S5. Association among the seven anthropometric indicators and hepatic steatosis in nonobese steatosis adults.

Outcome	Indicator (cut-off value)	Model1		Model2		Model3		Model4	
	**FMI1 (6.91)**	**3.14 (2.14-4.62)**	**<0.001**	**5.24 (3.08-9.22)**	**<0.001**	**5.00 (2.87-9.02)**	**<0.001**	**4.80(2.70-8.80)**	**<0.001**
	FMI2 (4.69)	2.35 (1.56-3.51)	<0.001	3.52 (1.93-5.93)	<0.001	2.91 (1.65-5.21)	<0.001	3.36(1.83-6.29)	<0.001
Nonobese steatosis	WWI (10.62)	5.98 (4.02-8.97)	<0.001	4.16 (2.64-6.61)	<0.001	3.56(2.20-5.81)	<0.001	3.36(1.83-6.29)	<0.001
（BMI<25）	BMI (21.65)	3.45 (2.24-5.44)	<0.001	2.91(1.83-4.73)	<0.001	2.62 (1.63-4.32)	<0.001	2.46(1.47-4.21)	<0.001
	BF% (24.7)	2.49 (1.60-3.98)	<0.001	4.13 (2.29-7.62)	<0.001	4.22(2.24-8.13)	<0.001	3.50(1.80-6.93)	<0.001
	WHR (0.89)	5.74 (3.86-8.59)	<0.001	3.52 (2.22-5.60)	<0.001	2.64 (1.61-4.34)	<0.001	2.31 (1.34-4.00)	<0.001
	ASMI (8.26)	0.23 (0.05-0.62)	0.013	0.28 (0.06-0.82)	0.041	0.29 (0.07-0.901)	0.057	2.52 (0.05-0.86)	0.047

Model 1: Crude model.

Model 2: Adjusted for age, sex, and race.

Model 3: Model 2 in addition to hypertension, diabetes, education level, smoking at least 100 cigarettes in one’s lifetime, alcohol consumption, and the family income to poverty ratio.

Model 4: Model 3 in addition to physical activity, hepatitis B or C, HDL cholesterol, albumin, globulin, ALT, AST, LDH, total bilirubin, and triglyceride levels.

Abbreviations: FMI1, fat mass index ([fat mass]/height^2); FMI2, fat mass index ([fat mass]/height^3); WWI, weight-adjusted-waist index; BMI, body mass index; BF%, percentage of body fat; WHR, waist-to-hip ratio; ASMI, appendicular skeletal muscle index.
